# Sealing the Leak: Percutaneous Closure of a Left Ventricular Pseudoaneurysm

**DOI:** 10.1016/j.jaccas.2025.104343

**Published:** 2025-07-30

**Authors:** Jeffrey Taylor, Joaquim Barboza, Khalil Ibrahim, Khaled Abdelhady

**Affiliations:** a Division of Cardiology, University of Illinois Chicago, Chicago, Illinois, USA; b Division of Cardiothoracic Surgery, University of Illinois-Chicago, Chicago, Illinois, USA

**Keywords:** complication, computed tomography, echocardiography, imaging, left ventricle, occlude

## Abstract

Left ventricular pseudoaneurysm (LVPA) is a rare but potentially life-threatening complication. Although surgical repair remains the gold standard, percutaneous closure has emerged as a less invasive and feasible option for high-risk surgical candidates. We present the case of a 76-year-old woman with 2 prior sternotomies for mitral valve repair and subsequent replacement, who presented with worsening dyspnea on exertion. A computed tomography scan performed to rule out pulmonary embolism led to the incidental discovery of an LVPA. We describe a percutaneous approach using multimodal imaging guidance for successful coiling and closure of the LVPA with an Amplatzer ventricular septal defect occluder device.

## Graphical Abstract

**Figure:**
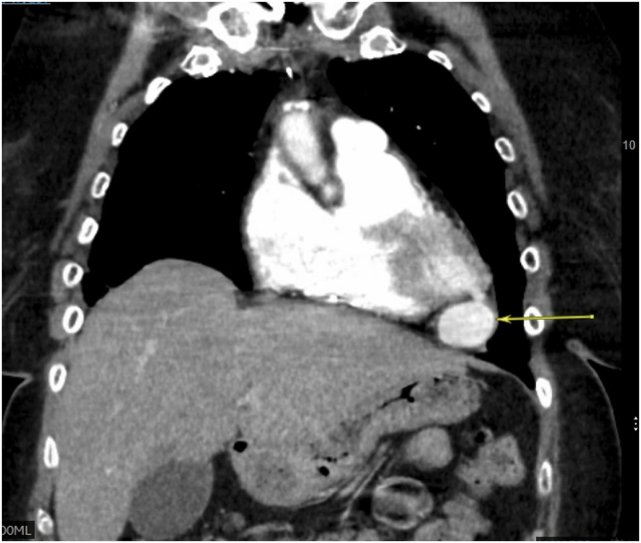

## History of Presentation

**Figure 1:**
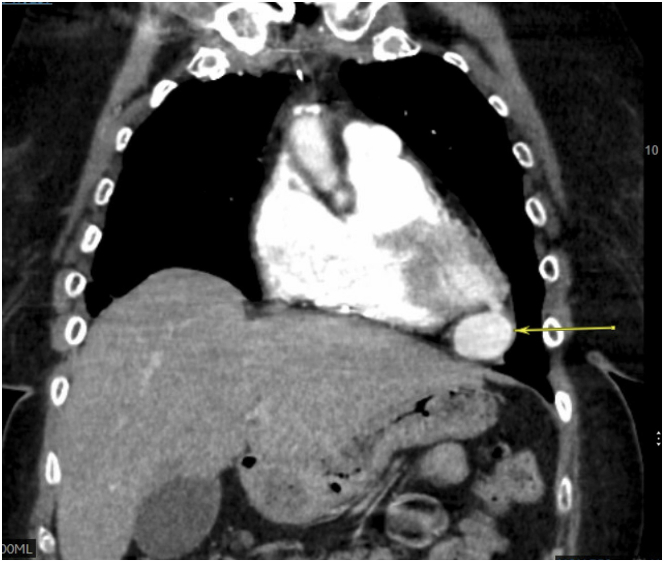
Computed Tomography Scan of the Chest Chest computed tomography with contrast enhancement demonstrates left ventricular pseudoaneurysm (arrow) at the apex of the left ventricle.

A 76-year-old woman presented to the emergency department with progressively worsening dyspnea over 1 month. Initial workup, including electrocardiogram, B-type natriuretic peptide, and laboratory tests, was unremarkable. She was in normal sinus rhythm, with no jugular venous distention, volume overload, or hemodynamic instability. Owing to her unexplained dyspnea, a computed tomography (CT) chest scan was performed, revealing a new left ventricular pseudoaneurysm (LVPA) (Figure 1), measuring 2.7 × 4.7 cm with a narrow neck of 0.6 cm.

**Take-Home Messages**

• LVPA is a rare but serious complication that requires timely diagnosis and intervention.
• Percutaneous closure is a viable alternative to surgical repair, particularly in high-risk patients.
• Multimodal imaging and a heart team approach are essential for optimizing patient outcomes.

## Past Medical History

• Hypertension
• Type 2 diabetes mellitus
• Hyperlipidemia
• Heart failure with preserved ejection fraction
• Severe mitral regurgitation
• Surgical mitral annuloplasty in 2002
• Bioprosthetic mitral valve replacement in 2024
• Atrial flutter
• Chronic kidney disease

## Differential Diagnosis

• LVPA
• Pericardial effusion
• Cardiac tamponade
• Infective endocarditis

## Investigations

A transthoracic echocardiogram with contrast enhancement confirmed the presence of the pseudoaneurysm (Video 1). Cardiac magnetic resonance provided further characterization of the morphology and wall integrity of the aneurysm. To facilitate procedural planning, coronary angiography and left ventriculography were performed, confirming an apical pseudoaneurysm with normal coronary arteries (Video 2). A multidisciplinary heart team discussion was held for treatment planning. Owing to the patient's multiple comorbidities and history of 2 previous cardiac surgeries, the patient was found to be a better candidate for a percutaneous approach rather than an open surgical approach.

## Management

The patient underwent antegrade percutaneous closure with transesophageal echocardiography and fluoroscopy guidance. Access was obtained via the femoral vein and radial artery. After a transseptal puncture with a Baylis radiofrequency needle (Baylis Medical), an Agilis steerable sheath (Abbott) was advanced across the atrial septum. Over a J-wire, a multipurpose catheter was introduced into the left ventricle and then into the pseudoaneurysm (Video 3). This was exchanged for an Amplatz Super Stiff (Boston Scientific) wire. A TorqVue2 delivery sheath (Abbott) was advanced into the pseudoaneurysm. Framing coils (Axium 25 mm × 50 cm [Medtronic] and Concierto 18 mm × 40 cm [Medtronic]) and 4 helical packing coils were deployed within the pseudoaneurysm (Video 4). Finally, an Amplatzer 10-mm muscular ventricular septal defect occluder device (Abbott) was deployed at the pseudoaneurysm neck, achieving successful closure (Video 5).

## Outcome and Follow-Up

Postprocedural left ventriculography (Video 6A) and transthoracic echocardiography (Video 6B) showed near-total control of the LVPA with a small residual leak, which was completely sealed at 1-month follow-up (Video 7). The patient tolerated the procedure well and was discharged 2 days later with no complications.

## Discussion

LVPA is a rare but potentially life-threatening complication.^1^ Although surgical repair remains the gold standard, percutaneous closure has emerged as a less invasive and feasible option for high-risk surgical candidates.^2^ While no specific guidelines exist for LVPA management, surgical repair remains the preferred approach. However, surgical closure carries a high mortality rate (20%-36%),^3^ with conservative management yielding even worse outcomes (48%).^4^ Percutaneous approaches have emerged as viable alternatives, particularly in high-risk surgical patients.^5^ Our patient's history of 2 prior sternotomies placed her at elevated risk for a third sternotomy. The likely etiology of her pseudoaneurysm was iatrogenic, related to prior left ventricular venting or wire perforation during mitral valve surgery.

Multimodal imaging (CT, cardiac magnetic resonance, and echocardiography) was instrumental in preprocedural planning, ensuring appropriate device selection and procedural success. Intraoperative transesophageal echocardiography and fluoroscopy facilitated precise device deployment, highlighting the importance of imaging in complex structural heart interventions.^6^

## Conclusions

This case adds to the growing body of literature supporting percutaneous LVPA closure as a safe and effective alternative to surgical repair in selected patients. Multimodal imaging and a collaborative heart team approach are critical to optimizing procedural success and patient outcomes.^7^

## Funding Support and Author Disclosures

The authors have reported that they have no relationships relevant to the contents of this paper to disclose.

## Appendix

Video 1 Transthoracic Echocardiography With Contrast Demonstrates Flow of Contrast From Left Ventricle to Pseudoaneurysm

Video 2 Left Ventriculography With 6-F Pigtail MIV Catheter Demonstrates Contrast Flow Into the Pseudoaneurysm

Video 3 Agilis Steerable Sheath in Place Across the Interatrial Septum With Multipurpose Catheter Being Advanced Over a J-Wire Into the Pseudoaneurysm

Video 4 A TorqVue2 Delivery Sheath Advanced Into the Pseudoaneurysm Framing coils (Axium 25 mm × 50 cm and Concierto 18 mm × 40 cm) were deployed within the pseudoaneurysm.

Video 5 Amplatzer 10 mm Muscular ventricular Septal Defect Occluder Device Deployed at the Pseudoaneurysm Neck

Video 6 (A) Post-procedural left ventriculography showed near total control of the LVPA with a small residual leak. (B) TTE post-procedure demonstrates absence of echo contrast flow through the pseudoaneurysm. (A) Post-procedural left ventriculography showed near-total control of the left ventricular pseudoaneurysm with a small residual leak. (B) Transthoracic echocardiography post-procedure demonstrates the absence of echo contrast flow through the pseudoaneurysm..7

Video 7 One-Month Post-Procedure Follow-Up Left Ventriculogram Demonstrates Complete Closure of Pseudoaneurysm8
